# Bioactive Peptides in Greek Goat Colostrum: Relevance to Human Metabolism

**DOI:** 10.3390/foods13233949

**Published:** 2024-12-06

**Authors:** Maria Louiza Petre, Anna Nefeli Kontouli Pertesi, Olympia Eirini Boulioglou, Eleana Sarantidi, Artemis G. Korovesi, Athina Kozei, Angeliki I. Katsafadou, George T. Tsangaris, Antonia Trichopoulou, Athanasios K. Anagnostopoulos

**Affiliations:** 1 Department of Biotechnology, Biomedical Research Foundation of the Academy of Athens, 11527 Athens, Greece; 2 Instituto Gulbenkian de Ciência, 1495 Oeiras, Portugal; 3 Faculty of Public and One Health, University of Thessaly, 43100 Karditsa, Greece; 4 Center for Public Health, Research and Education, Academy of Athens, 11528 Athens, Greece; 5 Oncology Unit, 3rd Department of Internal Medicine, “Sotiria” Hospital, Medical School, National Kapodistrian Univeristy of Athens, 11527 Athens, Greece

**Keywords:** colostrum, metabolism, proteomics, bioactive peptides

## Abstract

Colostrum is essential for the survival and development of newborn mammals. This primary source of nourishment during the first days of infant life is rich in functional components conductive to the enhancement of neonate immunity and growth. Compared with mature milk, a higher protein and peptide content is observed in colostrum, whilst it is low in fat and carbohydrates. The functional properties of colostrum are closely linked to the release of bioactive peptides during the gastrointestinal digestion of colostrum proteins. Our study aimed to comprehensively analyze the whey proteome of colostrum from indigenous Greek goats and to examine the influence of bioactive peptides released during digestion on human metabolism. Colostrum and mature milk samples from healthy ewes were subjected to nanoLC-MS/MS analysis, revealing differentially expressed proteins. These proteins were functionally characterized and subjected to in silico digestion. Using machine learning models, we classified the peptide functional groups, while molecular docking assessed the binding affinity of the proposed angiotensin-converting enzyme (ACE)- and dipeptidyl peptidase IV (DPPIV)-inhibitory peptides to their target molecules. A total of 898 proteins were identified in colostrum, 40 of which were overexpressed compared with mature milk. The enzymatic cleavage of upregulated proteins by key gastrointestinal tract proteases and the downstream analysis of peptide sequences identified 117 peptides predicted (with >80% confidence) to impact metabolism, primarily through modulation of the renin–angiotensin system, insulin secretion, and redox pathways. This work advances our understanding of dietary bioactive peptides and their relevance to human metabolism, highlighting the potential health benefits of colostrum consumption.

## 1. Introduction

Milk is a superior source of nourishment, rich in functional components including proteins, peptides, fats, vitamins, and minerals [1,2]. It is the chief source of nutrition during the first years of an infant’s life, supporting the dietary requirements of the developing organism [3,4,5]. In particular, colostrum, the first secretion of the mammary gland following labor, plays a key role in newborn mammal survival and development [6,7]. In comparison to mature milk, a lower fat and carbohydrate content is present in colostrum, which is rich in proteins and peptides conductive to the immunity enhancement and growth of the neonate [8,9,10,11,12].

The functional properties of colostrum are correlated to the existence of bioactive peptides inherently present within its proteins [13,14]. Bioactive peptides are short amino acid sequences, 2 to 20 residues in length, acting as modulators of physiology once released from their parent proteins [13,15]. In the gastrointestinal tract, this occurs via the enzymatic cleavage of the proteins by digestive proteases (e.g., pepsin, pancreatic enzymes) [16,17]. Of key importance is the capacity of bioactive peptides to exert a regulatory role on human metabolic processes such as glucose or redox metabolism [18,19,20,21]. A number of studies based on in silico, in vitro, and in vivo protein digestion verify the potent activities exerted by bioactive peptides. Crucially, these have been strongly correlated with the peptides’ capacity to withstand the digestive process, thereby entering the systemic circulation intact [22,23,24,25].

Animal-derived colostrum, when introduced in human nutrition may promote health benefits that are linked to its unique synthesis. To date, the potential therapeutic effects of colostrum, primarily of bovine origin, have been noted with regard to several aspects of human pathology, such as the amelioration of clinical outcomes in inflammatory or neurodegenerative diseases (e.g., inflammatory bowel disease, Parkinson’s disease) [26,27,28,29]. Moreover, studies have proposed the use of this material as a performance improving agent in athletes [30]. Nevertheless, significant knowledge gaps exist regarding the molecular mechanisms driving the health promoting effects of colostrum [31,32], particularly that originating from small ruminants, such as goats [33]. Despite the unique functional properties of goat milk and its higher digestibility and reduced allergenic potential in comparison with bovine milk [34,35,36], the proteome of caprine colostrum remains mostly uncharacterized. Variations in colostrum synthesis between indigenous and cosmopolitan goat breeds have been noted [37], and, to our knowledge, the proteome of Greek goat colostrum has not been described to date.

The present study aimed to generate a detailed portrait of the proteins found in the whey of Greek goat colostrum, identify the bioactive peptides inherently present within their sequences, and examine their influence on human metabolism. Machine learning algorithms and molecular docking were critically employed in the process of bioactive peptide functional annotation as well as in the attribution of their relevance to human metabolic processes. This work is set to advance knowledge on the physiological role of dietary bioactive peptides, concurrently unveiling the potential health benefits stemming from goat colostrum consumption.

## 2. Materials and Methods

### 2.1. Sample Collection and Preparation

Colostrum samples (N = 17), approximately 15 mL in volume, were collected from clinically healthy dairy does within 5 days following parturition. In detail, the colostrum samples were collected by hand expression from both mammary glands throughout the same lactation period. To reduce variability with regard to colostrum composition, samples were obtained from animals of the same age, nutritional status, and parity. Additionally, matched paired samples of mature milk (N = 17) were procured from this group of goats within the same lactation period. Specifically, approximately 15 mL of milk was aseptically collected from the animals, during milking. All the samples were frozen immediately upon collection and stored at −80 °C until further processing.

The frozen samples were completely thawed and centrifuged at 3500 rpm for 1 h, maintaining a temperature of 12 °C. The colostrum and the mature milk were separated into three distinct components, specifically, fat, milk serum (whey), and caseins. The milk serum (whey) fraction was retained, while the fat layer and casein precipitates were discarded. Subsequently, the whey proteins from the colostrum and mature milk were extracted and prepared for proteomic analysis.

### 2.2. Proteomic Workflow

#### 2.2.1. Protein Extraction

The colostrum and milk samples were precipitated with three volumes of acetone (1/3 *v*/*v*) and incubated overnight at −20 °C. Accordingly, a constant amount of sample (~350 μL), corresponding to the extracted whey fraction, was precipitated in approximately 1 mL of acetone. Next, the samples were centrifuged at 4000 rpm, 4 °C, for 30 min to discard the supernatant. The protein condensed precipitate was vacuum-dried in a SpeedVac apparatus (Eppendorf, Hamburg, Germany) for 45 min, shredded, and dissolved in a lysis buffer [4% *w*/*w* SDS, 0.1 M Tris-HCL, 0.1 M dithioerythritol (DTE) pH = 7.6]. To ensure thorough cell lysis, the samples were sonicated for 3 cycles of 12 s each at 38% amplitude using a tip sonicator (Vibra cell VC 505/VC 750, Newtown, CT, USA). The lysed cell solution was then centrifuged at 13,000 rpm, room temperature (RT), for 10 min, and the cell lysate supernatant was collected. The protein concentration of the supernatant was determined using the Bradford assay.

Subsequently, approximately 200–500 μg of the sample was transferred to an Amicon Ultra 0.5 Centrifugal Filter Device (Merck, Darmstadt, Germany) and centrifuged at 13,000 rpm, RT, for 15 min following the addition of an 8 M urea solution. This procedure was repeated once more, after which the proteins were alkylated by iodoacetamide (0.05 M iodoacetamide in 8 M urea, 0.1 M Tris-HCL pH = 8.5) at RT for 30 min in the dark. Finally, overnight digestion was carried out with the addition of trypsin (Roche Diagnostics GmbH, Mannheim, Germany) at a 1:100 enzyme-to-protein ratio [38].

#### 2.2.2. Peptide Separation and Tandem Mass Spectrometry Analysis

The digested peptides were redissolved in 70 μL of a phase A buffer (0.1% formic acid in bi-distilled water) prior to separation by high-performance liquid chromatography. The peptide separation was performed using a reverse-phase analytical C-18 column (75 μm × 50 cm; 100 Å, 2 μm-bead-packed Acclaim PepMap RSLC, Thermo Scientific) of an Ultimate-3000 system (Dionex, Thermo Scientific, Bremen, Germany), directly coupled to an LTQ-Velos Orbitrap Elite mass spectrometer (Thermo Scientific, Waltham, MA, USA). Regarding the liquid chromatography analysis, 6 μL of the peptide sample was injected into a C-18 precolumn (100 μm × 2 cm; 100 Å, 5 μm-bead-packed Acclaim PepMap 100, Thermo Scientific) at a constant flow rate of 5 μL/min and eluted in a phase A buffer. This was followed by a gradient of 2–33% phase B (99.9% acetonitrile, 0.1% formic acid), for a total elution time of 240 min, at a constant flow rate of 300 nL/minute.

The mass spectra were obtained using an Orbitrap Elite mass spectrometer equipped with a nanolectrospray source (Thermo Scientific, Waltham, MA, USA). The mass spectrometer was programmed to operate in a data-dependent acquisition mode, and the top 20 most intense ions were selected for fragmentation by high-energy collision dissociation (HCD) with a collision energy of 26%. Full-scan MS data were acquired at a resolution of 60,000, setting the mass range between 250 and 1250 *m*/*z*, while the MS/MS spectra were analyzed with a 15,000 resolving power as previously described [39]. The mass spectrometry instrument settings were configured using the XCalibur^TM^ software (v.2.2 SP1.48, Thermo Scientific, Waltham, MA, USA).

The raw data were processed with Protein Discoverer (version 1.4.0388, Thermo Scientific, Waltham, MA, USA). Protein identification was performed by searching against the UniProt Ruminantia, Carpa Prisca, and Ovis aries proteome databases using the SEQUEST tool. The database search criteria were defined as follows: up to two missed cleavages were allowed for trypsin digestion; the oxidation of methionine was set as a variable modification; and a 0.05 fragment ion tolerance and a 10 ppm peptide mass tolerance were set. To validate peptide spectral matches, a false discovery rate (FDR) of 1%, based on q-values, was applied. The minimum accepted length for peptide identification was 6 amino acids [40]. The obtained list of proteins was exported as a spreadsheet for further bioinformatic analysis.

### 2.3. Data Analysis

For each UniProt accession number corresponding to the identified colostrum and mature milk proteins, the best matching NCBI gene identifiers were retrieved using the ID mapping tool of the same database. The colostrum proteins were functionally annotated via Gene Ontology and the Kyoto Encyclopedia of Genes and Genomes (KEGG) pathway enrichment analysis, using the clusterProfiler (version 4.12.0) software package [41]. Gene annotations specific to *Capra prisca* were retrieved with the AnnotationHub (version 3.12.0) R tool [42]. The results were visualized in RStudio (version 2024.09.0+375) using the ggplot2 software package [43,44].

#### 2.3.1. Comparative Analysis (Colostrum and Mature Milk)

Quantitative proteomic data from the colostrum and mature milk samples obtained via label-free quantification (LFQ) methods were imputed using MICE (Multivariate Imputation by Chained Equations, v3.16.0) [45]. The differential expression of proteins in colostrum compared with mature milk was quantified via differential expression analysis (DEA), performed with DESeq2 (version 1.40.2) [46]. Proteins considered differentially expressed, based on strict criteria, |log2FoldChange| > 1, p-adjusted < 0.05, were functionally assigned to their corresponding biological pathways and mapped to KEGG pathways, using the clusterProfiler (version 4.12.0) and enrichplot (version 1.24.4) software packages [41,47]. The distinctive protein–protein interaction (PPI) networks formed by the upregulated and downregulated colostrum proteins were explored and visualized using the StringDB database (string-db.org) (version 12.0) [48].

#### 2.3.2. Protein Digestion and Peptide Functional Annotation

A simulated protein GI digestion model, based on key enzymes of the digestive tract, was employed. In silico proteolysis of the colostrum proteins was carried out with pepsin (EC 3.4.23.1), trypsin (EC 3.4. 21.4), and chymotrypsin (EC 3.4.21.1) under optimal enzyme conditions, using the BIOPEP Enzyme(s) Action tool [49].

Subsequently, the generated peptides were filtered based on potential bioactivity to retain potentially active sequences. Specifically, an advanced machine learning algorithm was used to evaluate the peptide bioactivity by assigning each sequence a probability score ranging between 0 and 1 [50]. Peptides allocated a score >0.5 (deemed biologically active) were retained, and their specific bioactivities were investigated. The bioactive peptides were mapped to 9 functional classes using a state-of-the-art machine learning-based (deep neural network) predictor [51]. Specifically, each peptide was assigned a score equal to the probability of belonging to the antioxidant, ACE-inhibitory, dipeptidyl peptidase IV (DPPIV)-inhibitory, antimicrobial, antifungal, antibacterial, antiviral, opioid, or neuropeptide bioactivity categories. Peptides functionally annotated with a high confidence (>80%) to each of the bioactivity categories were further evaluated using specialized algorithms with regard to their theoretical activity [52,53,54,55]. All the resulting plots were generated in RStudio with the ggplot2 package [43,44].

Finally, the ACE-inhibitory and DPPIV-inhibitory peptides predicted with high confidence using specialized deep learning algorithms relative to their specific activity [53,54] were subjected to molecular docking. Molecular docking is a powerful computational tool applied in drug discovery to elicit the structural basis of small molecule interactions with potential therapeutic targets [56,57]. In this study, the peptide molecular structures were retrieved using the BioPep “SMILES” tool [49], while the ACE and DPPIV crystal structures were obtained from the Protein Data Bank (PDB) [58,59]. The molecular docking analysis was carried out via the SWISSDock web server utilizing AutoDock Vina (version 1.2.0) [60,61]. The results were visualized with the Structures molecular viewer software tool (Version 1.8 (2402.07.2355)) [62].

**Figure 1:**
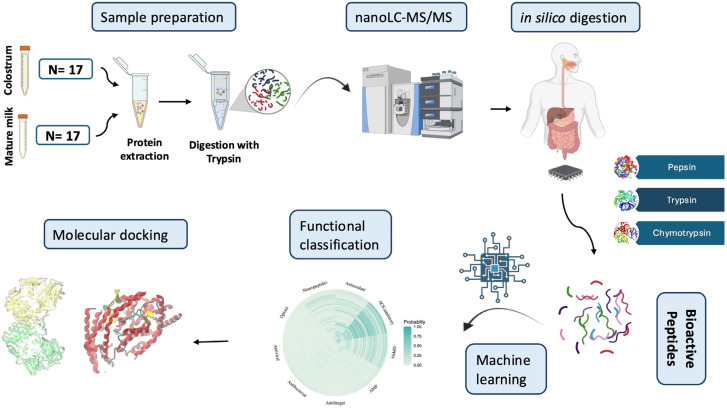
Workflow for the bioactive peptide annotation process in goat colostrum followed in this study. Samples were centrifuged, and whey proteins from colostrum and mature milk were subjected to nanoLC-MS/MS analysis. Following protein identification, a simulated protein GI digestion pipeline was employed. The obtained peptides were functionally annotated using advanced machine learning algorithms, and their binding affinity to selected target molecules (ACE, DPPIV) was evaluated using molecular docking.

The experimental design and computational workflow of this study are illustrated in Figure 1.

## 3. Results

### 3.1. Proteomic Landscape

In the present study, we generated the quantitative proteomic profiles of colostrum and mature milk from clinically healthy dairy does across the same lactating period using an untargeted LC-MS/MS approach. On average, a greater number of proteins were identified in colostrum samples than in mature milk. Applying a 1% false discovery rate (FDR) for quality control, we detected 898 and 539 proteins present in all colostrum and milk samples, respectively. Of these, a total of 245 proteins were common between samples collected during early (<5 d) and late lactation. The Greek goat colostrum proteins are presented in Supplementary Table S1.

**Figure 2:**
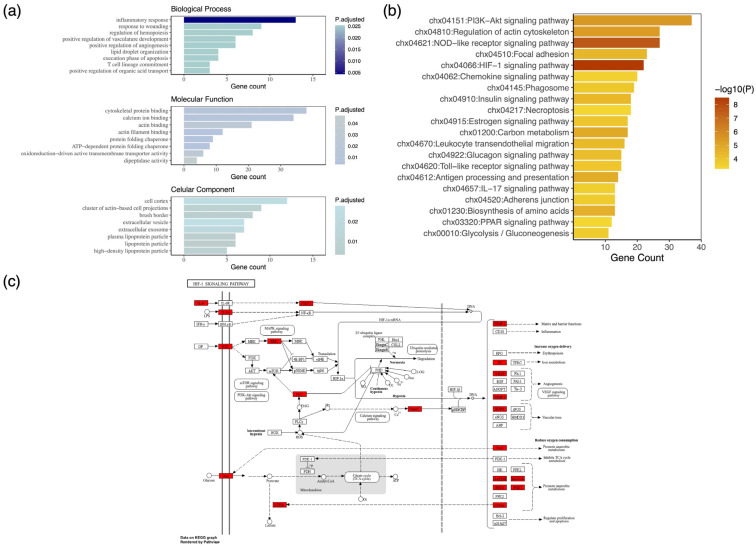
Proteomic characterization of goat colostrum whey proteins. (**a**) Gene Ontology (GO) analysis. Bar plot of the top statistically significantly enriched GO biological processes, cellular components, and molecular functions. (**b**) Biological pathway analysis. Bar plot of the top 20 enriched KEGG pathways. Colostrum proteins are the central components of mammary gland development and immune system processes. (**c**) Complete map of the HIF-1 signaling pathway, the most statistically significant molecular pathway of the identified proteins, depicted here in red.

To identify the functional properties of colostrum proteins, Gene Ontology enrichment analysis was carried out. The proteins were annotated with regard to their biological role, cellular distribution, and molecular function. As shown in Figure 2a, the proteins were significantly enriched for GO terms such as “inflammatory response”, “response to wounding”, and “regulation of hemopoiesis” in terms of their biological activity, in line with their crucial role in the establishment of immunity and maintenance of homeostasis in the newborn [63,64]. Thus, the proteins present in colostrum potentially safeguard healthy infant growth, ensuring survival. Notably, the above results reflect the indispensable function of colostrum toward the development of host defenses against exogenous parameters, including infectious agents. Furthermore, molecular binding (e.g., cytoskeletal protein binding, calcium ion binding, and actin filament binding) was significantly overrepresented with regard to colostrum proteins’ molecular function. Meanwhile, the proteins were primarily located in the cytoskeleton and extracellular space (e.g., cell cortex, extracellular vesicle).

Further, the biological pathways of the proteins identified in goat colostrum were elucidated. KEGG enrichment analysis revealed their central role in mammary gland development during early lactation, as well as in immune response in the infant. Indeed, the most statistically significant pathway was that of HIF-1 (hypoxia-inducible factor 1) signaling (Figure 2b), a key regulator of cellular adaptation and growth under hypoxic conditions [65]. Activated in response to hypoxia, HIF-1 is an evolutionarily conserved pathway, whose molecular targets are associated with cellular metabolism, proliferation, erythropoiesis, angiogenesis, and alterations in blood vessel structure [66,67]. Rapid development of the mammary gland, disproportionate to angiogenesis, may temporarily induce hypoxic conditions, thus activating HIF-1 [68]. Importantly, in mice, the deletion of HIF-1 has been linked to impaired lactation [69]. The complete network of the HIF-1 molecular pathway is illustrated in Figure 2c.

### 3.2. Comparative Analysis of Colostrum and Mature Milk

**Figure 3:**
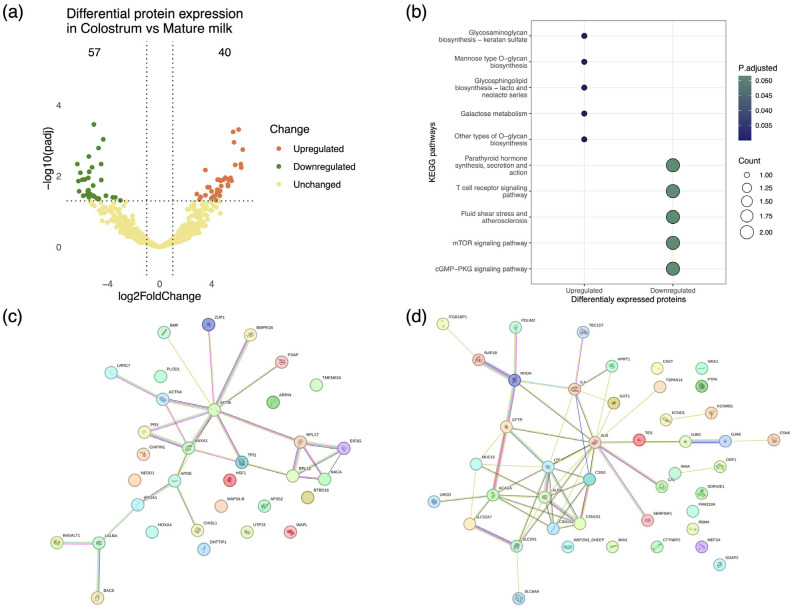
Proteomic variability associated with lactation stage in caprine mammary secretions from clinically healthy goats. (**a**) Volcano plot depicting protein differential expression in colostrum relative to mature milk. (**b**) Dot plot depicting the functional classification of differentially expressed proteins in colostrum and milk. Functional annotation of proteins performed in reference to the AnnotationHub *Capra hircus* KEGG annotation collection. (**c**,**d**) StringDB protein interaction networks representing pathways associated with upregulated and downregulated proteins in colostrum. Proteins are presented as color-coded nodes, and edges indicate protein–protein interactions.

Changes in milk composition during the course of lactation have been noted in most lactating species (e.g., cattle, sheep, and goats) [70,71,72]. We investigated proteome alterations occurring in caprine colostrum stemming from the transition to mature milk. The differential expression of proteins between the sample types was quantified by setting stringent criteria, specifically, log2FoldChange > 1 and *p*-value < 0.05, and the results were visualized via a volcano plot (Figure 3a). A total of 97 proteins were found to be statistically significantly differentially expressed between colostrum and mature milk, namely, 40 upregulated and 57 downregulated. The results revealed a significant enrichment of the protease inhibitor alpha-2 macroglobulin, which is known to regulate key inflammatory response pathways, in colostrum [73]. Additionally, we noted the upregulation of the MAP 34-A protein, an antimicrobial peptide and member of the cathelicidin family, possessing broad-spectrum antimicrobial activity [74]. The antimicrobial properties of MAP34-A have been associated with the inhibition of nucleic acid synthesis and the induction of oxidative stress in pathogenic microbes. Moreover, the secretion of this peptide in milk has been attributed to leukocytes and may actively contribute toward host defense via suppressing the production of pro-inflammatory cytokines [75,76]. All proteins found to be differentially expressed in colostrum relative to mature milk as determined by fold change are listed in Supplementary Table S2.

The differentially expressed proteins were functionally annotated by means of Kyoto Encyclopedia of Genes and Genomes (KEGG) enrichment analysis, showing a strong association of upregulated proteins in colostrum with immune response and newborn development [77,78], e.g., glycosaminoglycan biosynthesis, mannose type O-glycan biosynthesis (Figure 3b). To further delineate the role mediated by differentially expressed proteins, protein–protein interaction networks were constructed. The interactome formed by the upregulated proteins was composed of 33 nodes and 27 edges, while a network of 43 proteins connected by 55 interactions was formed by the downregulated proteins (Figure 3c,d).

The network of upregulated proteins was mainly driven by ACTB (beta-actin), ANXA 1 (annexin A1), and APOE (low-density apolipoprotein E). Importantly, these proteins are linked to essential biological structures/processes, e.g., cell structure, fatty acid synthesis, and lipid transport. Furthermore, the proteins albumin (ALB) and lactoferrin (LTF) were identified as being central to the interaction network of downregulated proteins.

#### Bioactive Peptides in Colostrum: Functional Classification and Relevance to Human Metabolism

In the present study, the release of bioactive peptides following colostrum digestion was exhaustively investigated, and their relevance to human metabolism was attributed based on their properties. Simulated GI protein digestion was carried out toward the discovery of functional peptides in goat colostrum, a protein-rich novel food. In total, 4346 proteolytic fragments of varying amino acid chain lengths (2-29 a.a.) were identified. The majority of discovered hydrolysates were di- and tripeptides and, thus, were likely to traverse the intestinal barrier [22], therefore entering systemic circulation.

**Figure 4:**
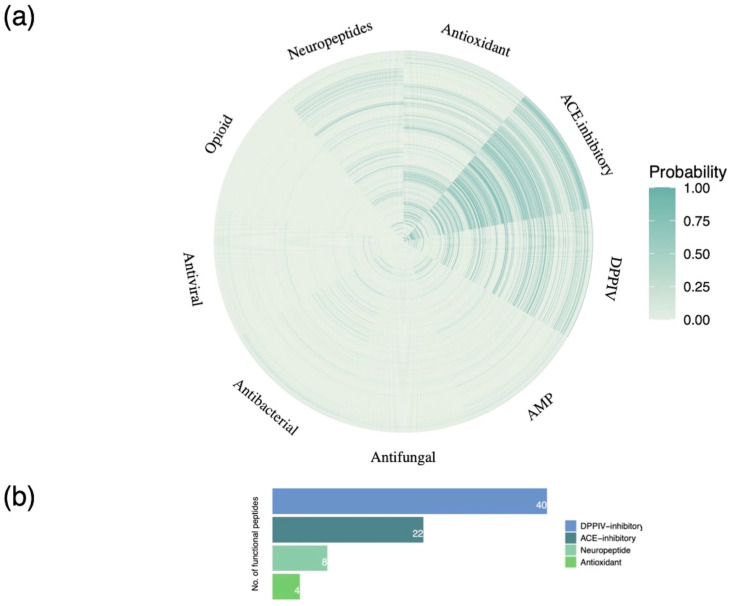
Functional classification of goat colostrum bioactive peptides. (**a**) Heatmap depicting confidence scores of 527 peptides assigned to 9 functional classes (antioxidant, antihypertensive, antimicrobial, antidiabetic, cardiovascular, neuropeptide, immunomodulatory, opioid, and celiac). (**b**) Bar plot showing the bioactivity categories of peptides predicted with high confidence.

The above pool of peptides was filtered on the basis of their potential bioactivity predicted using the Peptide Ranker tool, a state-of the-art deep learning model. Overall, a total of 527 peptides were assigned a probability score >50% in terms of general bioactivity and retained for downstream analysis. Peptide functional annotation was then carried out based on probability (confidence) scores generated by an advanced machine learning classifier. A confidence score was allocated to each peptide for nine functional classes representative of peptide bioactivity. The functional classification of the bioactive peptides inherently present in Greek goat colostrum proteins is presented in Figure 4a. Notably, the highest probability scores were recorded with regard to peptide ACE- and DPPIV-inhibitory, antioxidant, and neuropeptide-related activity. Importantly, a total of 177 peptides attributed these specific properties were characterized by a confidence score >80% in terms of bioactivity. Nevertheless, to further delineate the relevance of colostrum proteins to human metabolism, the abovementioned peptide sequences were further evaluated utilizing specialized algorithms with regard to their respective bioactivities. Consequently, 74 peptides displaying DPPIV and ACE-inhibitory, antioxidant, and neuropeptide-associated activity were discovered (Figure 4b).

### 3.3. Bioactive Peptide Screening by Molecular Docking

In the present study, the binding affinity of high-confidence predicted DPPIV-inhibitory and ACE-inhibitory peptides to their respective target molecules was evaluated by peptide docking. The inhibitory ratios of all peptides exerting potential DPPIV- and ACE-inhibitory activity is provided in Supplementary Table S3. The crystal structures of dipeptidyl peptidase IV (DPPIV) and angiotensin-converting enzyme (ACE) were retrieved from the Protein Data Bank [58,59], and docking analysis was performed via the SwissDock web server using AutoDock Vina [60,61]. The bioactive peptides displaying the most potent inhibitory activity against their respective molecular targets, based on molecular docking, were chosen for further analysis.

**Figure 5:**
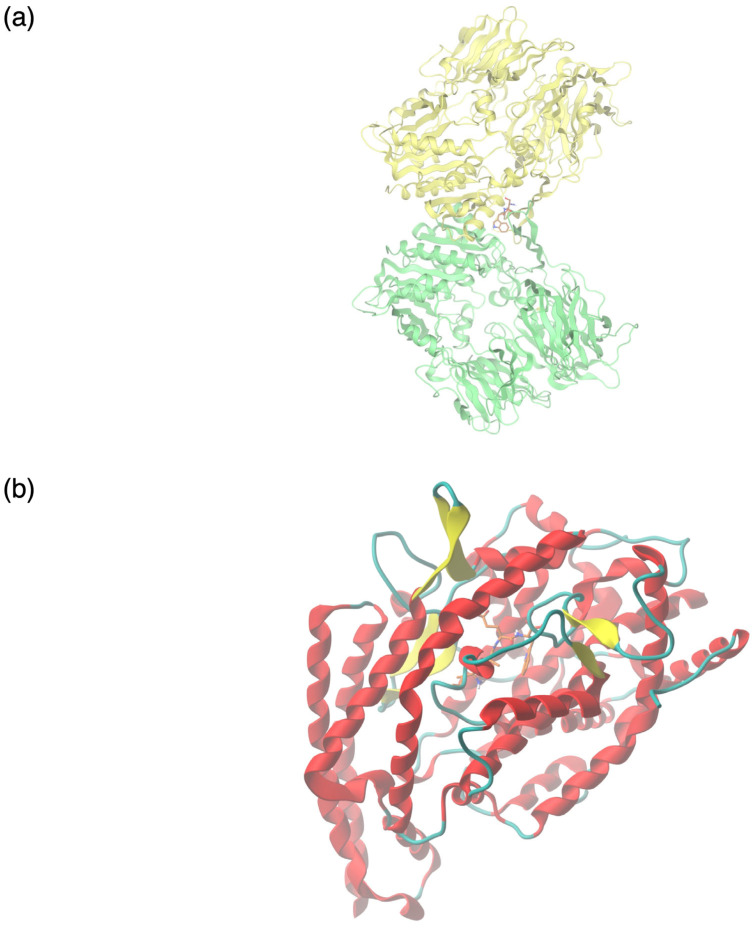
Molecular interactions between colostrum-derived bioactive peptides with predicted potent ACE- and DPPIV-inhibitory capacity and their respective targets. (**a**) Molecular binding of the dipeptide serine–tryptophan (SW) to the crystal structure of DPPIV (PDB ID 1NU6). (**b**) Molecular binding of the tetrapeptide isoleucine–alanine–glutamine–tryptophan (IAQW) to the crystal structure of ACE (PDB ID 1O8A).

The colostrum-derived peptides displayed variable binding affinities to DPPIV, ranging between −2.1 kcal/mol and −7.1 kcal/mol. The highest binding energy was shown by the dipeptide serine–tryptophan (SW) (Figure 5a) and was comparable to that of the DPPIV inhibitor alogliptin (−7.3 kcal/mol), which was used as a standard. The majority of the potentially ACE-inhibitory peptides examined displayed excellent binding affinity to their target molecule. Specifically, this was estimated as ranging between −5.28 kcal/mol and −9.365 kcal/mol. The tetrapeptide isoleucine–alanine–glutamine–tryptophan (IAQW) (Figure 5b) displayed the highest binding energy, −9.365 kcal/mol, approximately double that of the prescribed ACE inhibitor captopril (−5.23 kcal/mol) used as a standard.

## 4. Discussion

In this study, the presence of bioactive peptides in goat colostrum was investigated with a view to interrogate their relevance to human metabolism. The in-depth proteomic characterization of whey from Greek goat colostrum revealed the presence of 898 unique proteins, a substantial increase in identifications compared with previous investigations of caprine colostrum [11,79]. Additionally, the analysis of mature goat milk samples yielded a total of 539 unique proteins, in line with the study of Anagnostopoulos et al. [35], who reported the presence of 486 and 595 protein groups in the whey of two pure Greek goat breeds.

The colostrum proteins were annotated to existing Gene Ontology information regarding molecular function, biological processes, and cellular components. Specifically, based on Gene Ontology analysis, the observed proteins were primarily involved in “cytoskeletal protein binding” and “calcium ion binding”, therefore mediating tissue organization and cell signaling. Regarding their bioactivity, the proteins were primarily associated with immune system response, e.g., “inflammatory response” and “regulation of hemopoiesis”. With reference to cellular localization, the whey proteins from colostrum were classified as belonging to the “cell cortex” and the “extracellular vesicle”. Despite displaying a similar topology to proteins identified by [79], the proteins in the present study were primarily situated in the intracellular space. While recent studies have highlighted the role of caprine colostrum proteins in the glycolysis pathway and in disease-related processes [11,79], our results offer a novel outlook regarding their biological activity, molecular function, and pathway involvement.

It is noteworthy that the KEGG annotation and enrichment analyses highlighted the critical role of colostrum proteins in mammary gland physiology during early lactation (e.g., HIF-1 signaling pathway) [69]. Furthermore, the significance of colostrum proteins in facilitating the immune response (e.g., inflammatory response, chemokine signaling pathway) in the infant was highlighted. Colostrum supply is characterized as essential for the establishment of passive immunity transferred from the mother to the neonate, particularly in agammaglobulinemic animals such as goats but also in humans [6,63].

Proteins exert bioactivity following their cleavage by proteases (digestive, plant or microbial enzymes); therefore, the digestion of upregulated colostrum proteins was carried out using in silico tools [49]. Further, the bioactivity of the generated peptides was predicted using machine learning algorithms [50,51]. The in silico digestion of the protein sequences and the subsequent analysis of the digests, enabled by advances in bioinformatics, represent a sustainable pipeline for the discovery and characterization of bioactive peptides. The screening of prospective functional peptides is facilitated, enabling the selection of those displaying potent bioactivity for further in vitro or in vivo experimentation [80,81].

The presence of several ACE-inhibitory and DPPIV-inhibitory peptides in caprine colostrum was noted herein. Protein digestion was carried out utilizing core GI tract enzymes; the three main digestive proteases (gastric pepsin, trypsin, and chymotrypsin) were utilized for the cleavage of the protein sequences. The detected bioactive peptides, mostly 2–4 amino acid residues in length, were predicted with high confidence (>80%) to possess ACE-inhibitory, DPPIV-inhibitory, antioxidant, and neuropeptide properties. Evidence suggests that such small peptides may traverse the intestinal barrier intact, thus entering the blood stream and exerting their purposed biological activity [22,82,83].

The colostrum protein digests exhibited strong angiotensin-converting enzyme (ACE)-inhibitory potential, as revealed by the molecular docking analysis. Several small peptides docked against the ACE molecule showed increased binding affinity in comparison with the prescribed drug compound captopril based on their higher negative binding energies (ranging between −5.2 kcal/mol and −9.365 kcal/mol). In molecular docking, higher negative binding energy values are associated with an enhanced capacity of ligands to bind target molecules [84]. The strongest ACE-inhibitory activity was exerted by the tetrapeptide isoleucine–alanine–glutamine–tryptophan (IAQW), a precursor of the tripeptide IAQ, which has been shown to possess antihypertensive activity in vitro [85,86]. Interestingly, the presence of a hydrophobic amino acid at the C-terminus and the penultimate amino acid side chain size were linked to increased capacity regarding ACE inhibition utilizing Quantitative Structure Relationship Analysis (QSAR). Nevertheless, the role of peptide sequences toward their function requires further elucidation [87]. On the whole, due to the capacity of their inherently present peptides to act as ACE inhibitors, milk proteins are regarded as a chief source of peptides displaying antihypertensive properties. The fundamental mechanism by which ACE promotes an increase in blood pressure is characterized by ACE cleavage of the dipeptide HL, leading to the conversion of angiotensin I to the active form angiotensin II, a potent vasoconstrictor [88,89]. According to the available evidence, millions of people are impacted by hypertension, a known risk factor of cardiovascular and renal disorders [90], diseases linked to high morbidity and mortality. To this end, the optimized discovery of bioactive peptides, such as the workflow presented in this study, may provide a valuable tool toward the non-pharmacological amelioration of hypertension.

Another group of bioactive peptides, of particular interest, identified in caprine colostrum digests display a dipeptidyl peptidase IV (DPPIV)-inhibitory function as revealed by functional annotation and molecular docking. Dipeptidyl peptidase IV is a serine protease acting on glycemic control, by means of incretin hormone (GLP-1, GIP) degradation, thereby modulating insulin secretion patterns in response to food ingestion [91,92]. As impaired insulin secretion is a confounding factor in the appearance of type 2 diabetes, a chronic multifactorial disease with increased risk of vascular complications affecting millions of patients worldwide [93], food-derived bioactive peptides represent a promising adjuvant strategy in the context of disease management. In particular, the capacity to act on glucose metabolism has been demonstrated both in silico and in vivo by a diverse array of peptides released from casein and whey protein sequences present in goat milk (e.g., YPF, LLLP, MHQPPQPL, VMFPPQSVL, FNPTY, SPPEFLR) [94,95,96]. In this study, the most potent DPPIV-inhibitory activity was exerted by the dipeptide serine–tryptophan (SW), which exhibited improved binding affinity to its molecular target in comparison to the prescribed antidiabetic drug compound alogliptin based on its higher negative binding energy (−7.1 kcal/mol and −7.3 kcal/mol, respectively). Interestingly, the antidiabetic activity of this dipeptide has been previously observed by Lan et al. [97]. Moreover, our findings are further supported by those of Hrynkiewicz et al. [98], which showed the heightened capacity of food-derived dipeptides containing nonpolar amino acids to act as DPPIV inhibitors.

Furthermore, multiple dipeptides observed amongst the colostrum protein digests were predicted to act on oxidative metabolism. These include the dipeptide proline–histidine (PH), which was previously observed in vitro, following the enzymatic hydrolysis of bovine casein by microbial proteases [99]. It is worth noting that the capacity to regulate oxidative metabolism has been demonstrated by a diverse array of milk-derived peptides, from casein (e.g., LLY, SRYPSY, YIPIQ, AVPYPQR, KVLPVPEK, ARHPHPHLSFM, NPYVPR) and major and minor whey (e.g., ALPMHIR, GLDIQK) protein sequences [20,100,101]. Specifically, their primary mechanisms of action include the attenuation of reactive oxygen species (ROS) production [100,101] and the modulation of antioxidant enzyme expression patterns [20,102,103]. For instance, Nrf2-Keap1 metabolic pathway activation by peptides from milk and cheese proteins has been noted [98,99]. Peptides may trigger Nrf2 translocation to the nucleus, thus promoting the expression of NAD(P)H, SOD, and other cytoprotective enzymes regulated by Nrf2-Keap1 pathway activation [20,102]. It is, thus, evident that peptides released primarily from milk protein sequences exert a regulatory effect on the intricate network that constitutes redox metabolism, a cornerstone of cellular metabolism [104].

By using a novel pipeline for the efficient screening of proteomes representative of complex protein-rich foods for the release of functional peptides following their digestion, we have attested to the relevance of bioactive peptides to several aspects of human metabolism. The continued optimization of our proposed pipeline is set to provide a system-level understanding of bioactive peptides’ capacity to act on human pathophysiology, all the while providing further evidence of the health promoting effects associated with dietary protein consumption, particularly from dairy sources.

## Data Availability

The data presented in this study are available on request from the corresponding author. The data are not publicly available due to privacy restrictions.

## Supplementary Materials

The following supporting information can be downloaded at https://www.mdpi.com/article/10.3390/foods13233949/s1, Table S1: Colostrum proteome; Table S2: Differentially expressed proteins in colostrum relative to mature milk; Table S3: Inhibitory ratio of bioactive peptides against ACE and DPPIV based on molecular docking.
